# Conservation and Evolution of Wildlife in the Context of Climate Change and Human Population Growth

**DOI:** 10.3390/biology13060440

**Published:** 2024-06-17

**Authors:** Wenbo Liao, Lingsen Cao

**Affiliations:** Key Laboratory of Southwest China Wildlife Resources Conservation (Ministry of Education), China West Normal University, Nanchong 637009, China; ls_11242024@163.com

## 1. Introduction

Global climate change results in variations in morphological traits, resource competition, species diversity, physiological activity, genetic diversity, habitat use, distributional range, and conservation status in organisms [1,2,3,4,5,6,7,8,9,10,11,12,13,14,15,16,17,18]. There is evidence that one million species of plants and animals are experiencing extinction risk due to climate change [19]. According to the latest International Union for Conservation of Nature (IUCN) Red List, over 33.3% of all assessed species (42,100 species) are classified as threatened species [20]. The extinction risk for most species mainly results from anthropogenic activity, human population growth, environmental pollution, habitat loss, biological invasions, and climate disruption [1,21]. In particular, future global climate changes associated with anthropogenic activity and human population growth are regarded as a crucial factor in driving biodiversity loss because human activity and population growth promote habitat destruction and overexploitation of the endangered species [22]. Hence, many species are forced to change their present distribution ranges to adapt to variations in environmental conditions under future climate change [2].

Changes in species distribution patterns under climate change have indicated that while some species experience a contraction in their distribution ranges, others continue to expand it [10]. As a result of climate change, it is important to assess the potential distribution ranges for most vulnerable species [13,17]. Species distribution models (SDMs) can predict shifts in the potential distribution range of species under both current and future environmental conditions, presenting an encouraging approach to plan habitat recovery for endangered species. As an important species distribution model, the MaxEnt model has precision in predicting outcomes and has spatiotemporal capabilities of extrapolation [19]. For instance, the MaxEnt model has the advantages of robustness with limited data points, a low sensitivity to collinearity among variables, and the capacity to model intricate associations among variables [23]. Therefore, the MaxEnt model has widely been used in assessing species conservation status to forecast hotspots for the protection and restoration of ecology [19,22,23]. In addition, it is important to provide suggestions to the conservation manager by addressing the effects of future climate change on the potential distribution ranges of species using the MaxEnt model.

## 2. An Overview of Published Articles

Our aims for this Special Issue are to improve knowledge on the understanding of the responses of wildlife to environmental changes caused by climate change and human population growth. So far, five papers have published in this Special Issue in *Biology*. 

The article by Gao et al. (contribution 1) uses the random forest model based on the sites of occurrence of the Amur tigers (*Panthera tigris altaica*) and based on environmental variables to predict their potential habitats in Heilongjiang and Jilin provinces. The random forest model exhibits good prediction accuracy with the AUC value and the true skill statistic (TSS) value. Gao et al. find that the suitable habitats in the Amur tigers in the field account for 16.3% of the total study areas using the Youden index. The suitable habitats are mainly distributed in the southern part of the Laoyeling Mountains at the border between Jilin and Heilongjiang provinces, the Sino-Russian areas of the border of Huilin–Raohe in the eastern part of the Wanda Mountains, and the Yichun Forest areas in the Lesser Khingan Mountains. Gao et al. stress that the potential habitat areas of Amur tigers are small and severely fragmented, with a lack of connectivity between patches as prey potential richness is the most critical factor in shaping the distribution range of the tigers. The authors point out that habitat protection, restoration, and ecological corridor construction of the Amur tigers in this area should be strengthened to solve the serious threat of habitat fragmentation. The authors suggest that conservation managers in this area should work hard in terms of population conservation, habitat restoration, and the construction of ecological migration corridors in the Amur tigers in future.

The article by Liu et al. (contribution 2) is focused on studying the interspecific variation in egg and clutch mass associated with allometry, clutch size, parental care, nest predation, and lifespan among 22 sympatric bird species at an altitude of 3430 m to test the “egg-clutch trade-off” hypothesis. This research shows that the variation in egg mass is not explained by clutch size when controlling for allometric effects, contrasting the prediction of the egg–clutch trade-off hypothesis. However, the authors find that clutch size is positively linked to parental care, predation rate, and lifespan, while egg mass is only positively linked to parental care. This research stresses the importance of life-history theories that the reduced clutch size or mass can avoid higher predator risk and declined parental care to prolong adult lifespan. It also demonstrates that clutch size displays greater effects on parental care, nest predation, and lifespan compared to egg mass because of the significant reduction in energetic investment in smaller clutches. The authors claim that this research improves the understanding of how different factors affect egg size and clutch size in high-altitude birds and can provide for conservation suggestions for these high-altitude birds.

The third article published in this Special Issue by Jameel et al. (contribution 3) uses the MaxEnt model to estimate habitat suitability of the Western tragopan (*Tragopan melanocephalus*) in varying climate scenarios from the Western Himalayas. This research utilizes a total of 200 occurrence points, with 19 bioclimatic, 4 anthropogenic, 3 topographic, and a vegetation variable in species distribution models (SDMs). The authors reveal that the areas with high and moderate suitability for the Western tragopan are patchily distributed in the Western Himalayas while the continuous strips of highly suitable habitats are observed in the Kashmir region and the Annapurna region of Uttarakhand. The authors point out that the habitat suitability in the Western tragopan is likely to increase under future climate change. Meanwhile, the authors provide suggestions for future research on population collapses and other potential drivers of local extinction risk. In addition, the authors claim that a more effective management strategy in wildlife in the Western Himalayas should be considered to help with the reintroduction of suitable habitats in the *T. melanocephalus* population under the changing climate and ultimately aim to protect this endangered species.

In the fourth article, Haq et al. (contribution 4) focus on identifying the potential suitable microhabitats of the high-mountain-dwelling Himalayan goral (*Naemorhedus goral*) under climate change and human activities using species distribution models (SDMs). The authors employ a total of 81 species presence points, with 19 bioclimatic and 3 topographic variables in species distribution models. The authors detect that annual precipitation and altitude are the most influential drivers on the distribution range of *N. goral* in the MaxEnt model. The authors point out that the habitat suitability of the targeted species is likely to expand under future climate changes. This research reveals that the targeted species may shift northwards along an elevational gradient away from human settlements. The authors recommend that the finding benefits in helping the future planning, management, and sustainable use of available resources for the targeted species in the Himalayan region with climate change. 

The research from Bashir et al. (contribution 5) delves into the medicinal and cultural meaning of birds and mammals in the surrounding area of the Ayubia National Park, Pakistan. The authors find that the local people utilize 18 birds and 14 mammals to treat different diseases. This research shows that local people need to improve their ethno-ornithological and ethno-mammalogical knowledge, which benefits in the sustainable use of biological diversity in this park. The authors suggest that it is necessary to develop contemporary pharmaceutical research to guarantee the secure utilization of the presented practices, and local communities should protect medicinal animals. 

## 3. Conclusions

The compilation of five articles devoted to variations in the conservation and use of wildlife under climate change and human activity encompasses a range of research, reflecting the effects of the richness of climate change and human population growth on conservation status in wildlife. This research adopts different methods, ranging from qualitative approaches based on meetings and interviews to discuss the cultural and therapeutic value, to quantitative approaches using species distribution models to predict the suitable habitats of the endangered species under future climate changes. 

In terms of subject, assessing the suitable habitats of the endangered species under future climate change makes their mark in this Special Issue, with three articles focusing on this format. It is noted that while the discussion about the potential suitable microhabitats of two endangered species from the Himalayas is central to two of the articles, the remaining one is dedicated to the study of suitable habitats of the Amur tigers in Heilongjiang and Jilin provinces. This research adds new readings on the study of the suitable habitats of the endangered species under climate change, ultimately providing suggestions of population conservation and habitat restoration for conservation managers in the studied area. In addition to the three articles that focus on the suitable habitats of the endangered species due to climate change, another two articles focus on different subjects. This article (contribution 2) explores variations in egg and clutch mass associated with ecological factors in sympatric birds at high altitude and understands the mechanism of small clutches in birds due to the significant reduction in energetic investments. Although this research does not estimate the suitable habitats of high-altitude birds, due to climate change, it focuses on variations in phenomenon characteristics (e.g., egg and clutch size) linked to environmental conditions. In this way, the constant research centered on variations in morphological traits, habitat uses, distributional ranges, and conservation statuses in wildlife is confirmed, whether or not in terms of emerging new methods, requiring researchers to adapt to continuous changes. However, this work (contribution 5) in this Special Issue centers on the vernacular taxonomy and cultural and ethnopharmacological applications of birds and mammals, which are not suitable for the subject of wildlife conservation and evolution associated with climate change and human population growth.

As a note, we emphasize the particularity that all articles are based on the studies from Asian countries that are usually less emergent in the literature. Hence, these novelty findings emphasize the importance of this Special Issue about biodiversity conservation, allowing the reader to search for studied fields focusing on the assessment of the suitable habitats in regional contexts. 
